# Supplementary material for: Resolving the full spectrum of human genome variation using Linked-Reads
Source: Genome Res. 2019 Apr;29(4):635–45. doi: 10.1101/gr.234443.118 (PMC6442396; doi:10.1101/gr.234443.118)
Supplement: Supplemental Material [file supp_gr.234443.118_Supplemental_longranger-2.2.zip › longranger-2.2.tar.bz2!/lr2.2-public/lib/stan/cmdstan/stan/doc/api/html/namespacemembers_eval.html]

Stan: Namespace Members


|  |  |
| --- | --- |
|  | Stan  2.5.0  probability, sampling & optimization |


- Main Page
- Related Pages
- Namespaces
- Classes
- Files

- Namespace List
- Namespace Members

- All
- Functions
- Variables
- Typedefs
- Enumerations
- Enumerator


All Classes Namespaces Files Functions Variables Typedefs Enumerations Enumerator Friends Macros Pages

- TERM\_ABSF
  : stan::optimization
- TERM\_ABSGRAD
  : stan::optimization
- TERM\_ABSX
  : stan::optimization
- TERM\_LSFAIL
  : stan::optimization
- TERM\_MAXIT
  : stan::optimization
- TERM\_RELF
  : stan::optimization
- TERM\_RELGRAD
  : stan::optimization
- TERM\_SUCCESS
  : stan::optimization


---

[ Stan Home Page ]

*© 2011–2014,
Stan Development Team.*
